# Investigation of mitochondrial inner membrane ion conductance by planar lipid bilayer electrophysiology

**DOI:** 10.3389/fphys.2026.1782998

**Published:** 2026-04-20

**Authors:** Amrendra Kumar, Eleanora Margulis, Nelli Mnatsakanyan

**Affiliations:** Department of Cell and Biological Systems, Penn State College of Medicine, Hershey, PA, United States

**Keywords:** adenine nucleotide translocator (ANT), ATP synthase leak channel, mitochondria, patch-clamp, planar lipid bilayer electrophysiology

## Abstract

Mitochondrial ion channels are proteins of the inner and outer mitochondrial membranes that regulate ion flux and control various cellular processes, including calcium signaling, bioenergetic and metabolic functions, and cell death. Their precise regulation is essential to maintaining normal mitochondrial function and preventing pathological processes. Patch-clamp and planar lipid bilayer electrophysiology techniques have been used to measure ion flow directly across the membrane, thereby revealing the gating kinetics and pharmacological profile of ion channels in real time. Here, we describe a planar lipid bilayer electrophysiology approach for assessing mitochondrial ion channel conductance using mitochondrial inner membrane vesicles (IMVs). The comparative electrophysiology analysis between IMVs and purified mitochondrial proteins, ATP synthase, and the adenine nucleotide translocator (ANT), demonstrates that planar lipid bilayer electrophysiology is a robust tool for biophysical characterization of mitochondrial ion channels using IMVs. This approach is particularly valuable for investigating ion channel properties under controlled yet physiologically relevant conditions and for evaluating the direct modulatory effects of different pharmacological agents.

## Introduction

Mitochondrial ion channels play multifaceted roles in cellular physiology and pathology (Miyawaki et al., 2008; Colombini and Mannella, 2012; Patron et al., 2013; Bernardi et al., 2023). They regulate cellular energy conversion (ATP synthesis), reactive oxygen species (ROS) generation, and programmed cell death by controlling ion flux across the outer and inner mitochondrial membranes (Miyawaki et al., 2008; Colombini and Mannella, 2012; Patron et al., 2013; Bernardi et al., 2023). The key mitochondrial ion channels include voltage-dependent anion channels (VDAC) of the outer membrane (Colombini, 2004), and various channels of the inner membrane that regulate the Ca^2+^ flux [mitochondrial calcium uniporter, MCU (Kirichok et al., 2004)], K^+^ flux [mitoKATP, mitoBKCa channels (Inoue et al., 1991; Garlid and Paucek, 2003; Sanghvi et al., 2022; Szabo and Szewczyk, 2023; Szewczyk et al., 2025)], H^+^ flux [uncoupling proteins, UCP1-5 (Bertholet and Kirichok, 2022), adenine nucleotide translocase, ANT (Bertholet et al., 2022)] and Cl^-^ flux [chloride intracellular channels (Ponnalagu et al., 2016; Ponnalagu and Singh, 2017)]. These channels, along with the large conductance, non-selective pore of the inner membrane, the mitochondrial permeability transition pore (mPTP) (Petronilli et al., 1999; Mnatsakanyan et al., 2017; Bernardi et al., 2023), act as master regulators of cell life and death.

Electrophysiological studies are central to understanding ion channel function and regulation, and to the discovery of therapeutics for channelopathies and other ion channel-related diseases (Petronilli et al., 1989; Kinnally et al., 1991; Szabo and Zoratti, 1992; Zoratti and Szabo, 1995; Lohret et al., 1996; Kinnally et al., 2006; Priest et al., 2007; Dunlop et al., 2008). Several ion channels have been identified as key contributors to various mitochondria-associated pathologies using electrophysiological approaches (Kirichok et al., 2004; Szabo and Zoratti, 2014; Szabo and Szewczyk, 2023). Among these was the mPTP (Kinnally et al., 1989; Petronilli et al., 1989; Kinnally et al., 1992; Zorov et al., 1992; Petronilli et al., 1999), which remains among the most debated ion channels in bioenergetics, particularly with respect to its molecular nature and regulation.

Traditionally, the following two main approaches have been used to investigate mitochondrial ion channels: (i) patch-clamp recordings of intact mitochondria or mitoplasts (inner membrane preparations) (Kinnally et al., 1989; Petronilli et al., 1989; Szabo and Zoratti, 1992; Jonas et al., 1997; Pavlov et al., 2005; Mnatsakanyan, 2017; Bertholet, 2021; Neginskaya and Pavlov, 2023), and (ii) planar lipid bilayer recordings of purified proteins reconstituted into artificial membranes (Pavlov et al., 2005; Colombini, 2007; Rosencrans et al., 2021; Mnatsakanyan et al., 2022).

Patch-clamp electrophysiology of mitoplasts is a well-established technique that enables the assessment of ion channel function within their native lipid environment (Szabo and Zoratti, 1992; Kinnally et al., 1996; Neginskaya and Pavlov, 2023). This approach allows precise measurement of single-channel conductance, gating kinetics, and ion selectivity of mitochondrial ion channels and has been instrumental in advancing our understanding of mitochondrial ion transport mechanisms over the past few decades. Nevertheless, patch-clamp electrophysiology is constrained by several technical challenges, including limited recording stability, the small membrane surface area available for forming a high-resistance seal between the pipette and the cell, and the difficulty of precisely delivering pharmacological agents to either side of the membrane. In addition, maintaining a stable giga-ohm seal over extended recording periods can be technically demanding and highly dependent on operator’s skills.

In contrast, planar lipid bilayer electrophysiology monitors the behavior of ion channels at the single-molecule level in artificial membranes under controlled conditions (Zakharian, 2013; Zakharian, 2021). Bilayer recordings facilitate the rapid and selective application of ligands and inhibitors to both sides of the membrane via the *cis* and *trans* compartments of the bilayer cuvette (Zakharian, 2013; Zakharian, 2021). However, this methodology requires solubilization and purification of mitochondrial proteins with detergents, followed by reconstitution into artificial lipid bilayer membranes. As a result, the single-channel activity of an ion channel is assessed not in the native mitochondrial membrane and in the absence of potential interacting partners and regulatory factors present in native cellular contexts that may modulate the ion channel activity (Jiang and Gonen, 2012; Saponaro and Lolicato, 2022).

To bridge this methodological gap, we developed a reconstitution and channel-recording protocol to measure ion conductance of mitochondrial inner membrane vesicles (IMVs) using planar lipid bilayer electrophysiology. This approach combines the advantages of patch-clamp and lipid bilayer electrophysiology techniques, enabling measurements of mitochondrial ion channel activity under controlled conditions while preserving native protein-lipid interactions. IMVs, however, contain numerous mitochondrial proteins, which increases the likelihood of multiple channel insertions in the artificial lipid bilayer. Here, we used specific pharmacological inhibitors to dissect the recorded channel activities and assign them to specific channels. In this study, we focused on characterizing the ion channels involved in mPTP formation.

mPTP is a high-conductance megachannel of the mitochondrial inner membrane with a 1.5 nS peak conductance and 0.3 nS mean conductance activity (Petronilli et al., 1989; Kinnally et al., 1991; Petronilli et al., 1999). It is a voltage-gated ion channel activated by Ca^2+^ and inhibited by adenine nucleotides and some divalent or trivalent ions (Ba^2+^, Gd^3+^). It was initially identified in patch-clamp recordings of mitoplasts (Kinnally et al., 1989; Petronilli et al., 1989; Kinnally et al., 1991; Kinnally et al., 1992; Szabo and Zoratti, 1992; Szabo et al., 1992), and is implicated as a unique regulator of cell life and death. The brief, reversible openings of mPTP, known as flickering events, serve a physiological function by regulating ROS signaling and preventing hyperpolarization of the inner membrane (Petronilli et al., 1989; Szabo and Zoratti, 1992; Zoratti and Szabo, 1995). In contrast, prolonged and irreversible mPTP openings induce inner membrane depolarization, halting ATP production, followed by mitochondrial swelling and rupture, release of mtDNA and cytochrome c, and activation of cell death. Despite the crucial role of mPTP in cellular physiology and pathology, its exact molecular nature is still debated (Bernardi et al., 2023).

Initially, mPTP was proposed to form from inner mitochondrial membrane proteins, ANT and the phosphate carrier, and the outer-membrane protein, VDAC (Szabo and Zoratti, 1993; Szabo et al., 1993; Halestrap and Brenner, 2003; Kwong et al., 2014). The bovine and porcine F_1_F_O_ ATP synthase dimers (Giorgio et al., 2013; Carraro et al., 2014; Carraro et al., 2018; Guo et al., 2019; Urbani et al., 2019) or monomers (Alavian et al., 2014; Mnatsakanyan et al., 2019) were reported to have biophysical properties similar to those of mPTP in several recent studies (Alavian et al., 2014; Giorgio et al., 2017; Mnatsakanyan et al., 2019). The ATP synthase c-subunit ring was proposed and remains a strong candidate for forming the structural pore-forming component of mPTP (Pavlov et al., 2005; Bonora et al., 2013; Alavian et al., 2014; Azarashvili et al., 2014; Morciano et al., 2018; Mnatsakanyan et al., 2019; Neginskaya et al., 2019; Mnatsakanyan et al., 2022). The ATP synthase c-subunit leak channel (ACLC) is gated by voltage and forms a multi-conductance, Ca^2+^-sensitive channel with 1.5 nS peak conductance activity (Alavian et al., 2014; Mnatsakanyan et al., 2022). The application of a well-known mPTP modulator, Ca^2+^, during patch-clamp recordings of purified reconstituted mammalian ATP synthase increases the frequency of channel opening, whereas ATP inhibits it (Alavian et al., 2014; Mnatsakanyan et al., 2019). In addition, we have recently found that bedaquiline (BDQ), the FDA-approved compound for treating pulmonary multidrug-resistant tuberculosis (TB) (Mahajan, 2013*)*, modulates ACLC activity (Kumar et al., 2026). Although BDQ is a more potent inhibitor of the catalytic activity of *M. tuberculosis* ATP synthase (Preiss et al., 2015; Guo et al., 2021), it was also reported to bind at the interface between the a- and c-subunits of mammalian ATP synthase inhibiting its catalytic activity (Luo et al., 2020; Zhang et al., 2024). We have reported the dose-dependent inhibitory effect of BDQ on the ACLC activity of porcine heart ATP synthase (Kumar et al., 2026).

In this study the predominant channel recorded in planar lipid bilayer recordings of IMVs was a voltage-gated channel with high conductance activity (~1.1 nS). It was insensitive to bongkrekic acid (BKA) (Brustovetsky et al., 2002; Halestrap and Brenner, 2003; Neginskaya and Pavlov, 2023), a specific inhibitor of the ANT channel, and was fully inhibited by ATP and BDQ (Kumar et al., 2026), thus most likely corresponding to the ACLC. It closely resembled the channel activity of purified porcine and bovine heart ATP synthase, analysed by patch-clamp recordings of reconstituted proteoliposomes (Alavian et al., 2014; Mnatsakanyan et al., 2019) and the planar lipid bilayer system (Urbani et al., 2019; Kumar et al., 2025).

Another high-conductance voltage-gated channel recorded during planar lipid bilayer studies of IMVs was selectively inhibited by BKA, consistent with properties of the channel formed by purified ANT reported in this and other published studies (Brustovetsky and Klingenberg, 1996; Brustovetsky et al., 2002).

Together, these findings suggest that the IMV reconstitution approach into planar lipid bilayers provides a robust platform for the mechanistic examination and pharmacological characterization of established ion channels and for dissecting the molecular identity of multipore channel complexes. In addition, this methodology could serve as an exploratory platform for discovering novel mitochondrial ion channels that conventional approaches might otherwise obscure.

## Materials and methods

### Mitochondria isolation and IMV preparation

**Figure 1:**
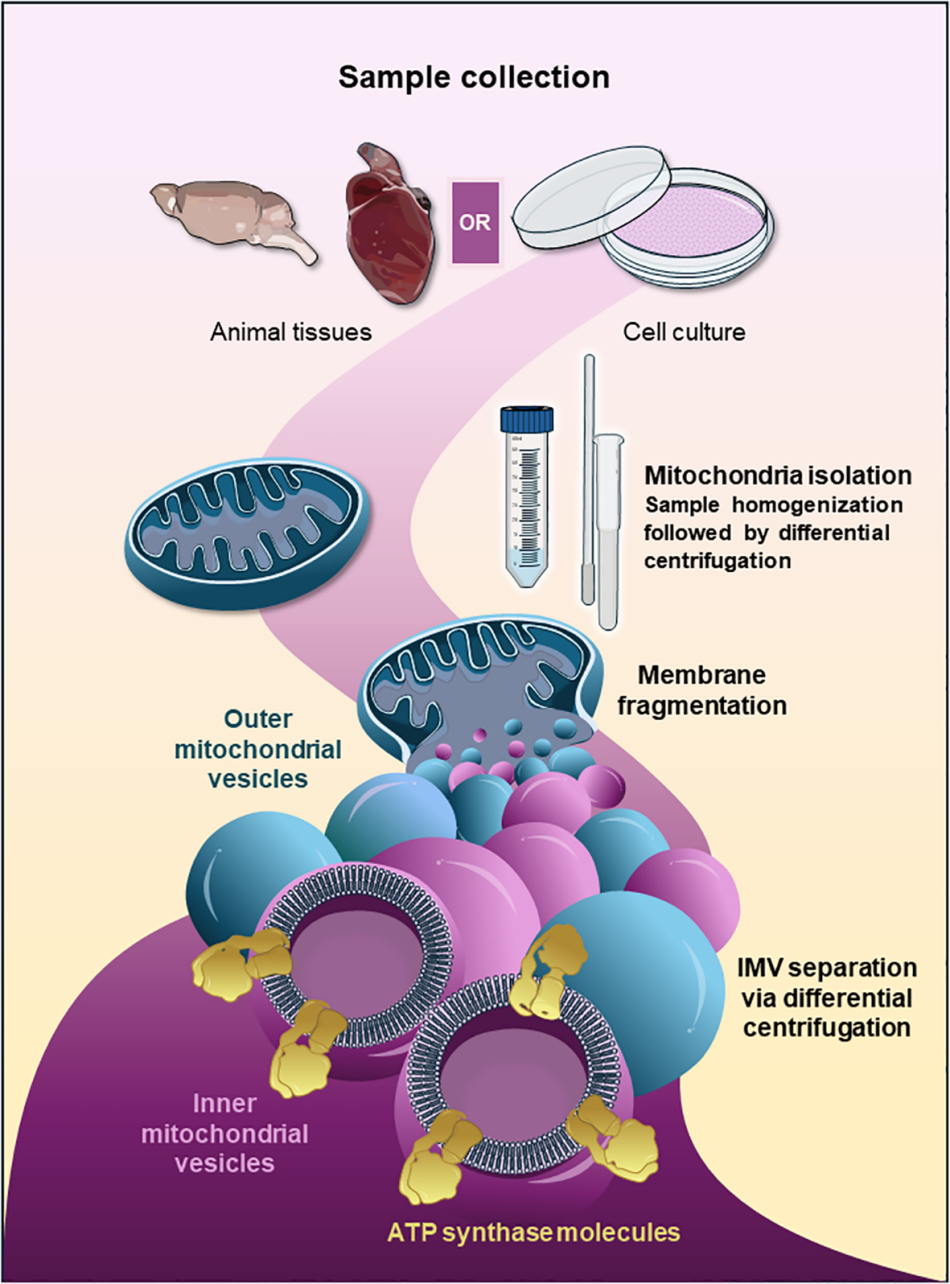
Schematic representation of the procedures for mitochondria isolation and IMV preparation. Mitochondria isolation procedure from freshly extracted animal tissues or cell cultures using the Potter-Elvehjem homogenizer, followed by centrifugation steps as described in Methods. Mitochondrial inner membrane vesicles (IMVs) enriched with ATP synthase are prepared by treating mitochondrial pellets with digitonin, followed by differential centrifugation to separate mitochondrial outer membrane vesicles.

**Figure 1** shows a schematic representation of the procedures for mitochondrial isolation and IMV preparation. Fresh porcine hearts were obtained immediately after slaughter, and the heart was finely minced in Isolation Buffer I (225 mM mannitol, 70 mM sucrose, 1 mM EGTA, and 20 mM Tris (pH 7.2)). The tissue was then homogenized with the Potter-Elvehjem, followed by centrifugation at 1000 x g for 4 min (**Figure 1**). The supernatant was transferred into a fresh tube, and the sediment was homogenized for a second time to access intermyofibrillar mitochondria. After centrifugation at 1000 x g, the supernatants were centrifuged at 9000 x g for 10 min to sediment the mitochondria. The mitochondria were resuspended in Isolation Buffer II (225 mM mannitol, 70 mM sucrose, and 20 mM Tris (pH 7.2)) and centrifuged again at 9000 x g for 10 min. Isolated mitochondrial pellets were quantified and either used immediately for IMV preparation or snap-frozen and stored at -80 °C until further use.

IMVs were prepared by modifying the previously published protocols (Chan et al., 1970; Sacchetti et al., 2013) (**Figure 1**). The isolated porcine heart mitochondrial pellets (4 mg/ml) were resuspended in Isolation Buffer III (250 mM sucrose, 20 mM HEPES, 1mM EDTA, 0.5% BSA, pH 7.4). 1% digitonin was added to the resuspended mitochondrial pellet, and the mixture was incubated on ice for 15 min. The mixture was then centrifuged at 16,000 x g for 10 min at 4 °C. The pellet was resuspended in Isolation Buffer I and then centrifuged again. The resulting pellet was again resuspended in Isolation Buffer I containing 0.1% Lubrol and incubated on ice for 15 min. This suspension was carefully layered onto the Isolation Buffer III and centrifuged at 182,000 x g for 60 min at 4 °C. The pellets were resuspended in Isolation Buffer IV (20 mM HEPES, 1 mM EDTA, 10% ethylene glycol, pH 7.4) and incubated at 4 °C for 30 min, then centrifuged at 30,000 x g for 15 min. The supernatant was collected and centrifuged at 182, 000 x g for 1h. The pellet containing IMVs enriched with ATP synthase was either used immediately or stored in liquid N_2_ until further use. The quality of isolated IMVs and the functional activity of ATP synthase were assessed before using them in any downstream applications (**Figure 1**; **Supplementary Figures S1a–c**).

### Purification of ATP synthase and ANT proteins

Mitochondria isolated from porcine heart were used for ATP synthase purification. Mitochondria were solubilized on ice by using the non-ionic detergent n-dodecyl-β-D-maltoside (DDM) (1 g/g protein) for 1h. The suspension was then centrifuged at 100,000 × g for 1 h. Subsequently, 50% (w/w) PEG 6000 was added to the supernatant (final concentration, 7%). After 2h of incubation on ice, the protein precipitate was removed by centrifugation at 50,000 × g for 1h, and PEG 6000 was subsequently added to the supernatant (final concentration, 2%). The protein precipitate was collected after incubation on ice for 2h, then centrifuged at 50,000 × g for 1 h. The pellet containing ATP synthase was solubilized in Buffer A (50 mM Tris-HCl, 100 mM NaCl, 2 mM MgSO4, 1 mM ATP, 0.05% DDM, pH 7.0 or pH 8.0). The solubilized sample was concentrated to 0.5 ml (Amicon centrifugal filters with 100 kDa molecular mass cutoff) and loaded onto a Superose 6 Increase 10/300 gel filtration column (GE Healthcare, USA) equilibrated with Buffer B (50 mM Tris-HCl, 150 mM NaCl, 0.05% DDM, pH 7.3). Eluted fractions corresponding to ATP synthase were collected and used for further analysis.

The mouse ANT1 (Slc25a4) construct, tagged with Myc and DDK (Flag) (Origene Technologies), was used for protein overexpression and purification. The construct was expressed in HEK293 cells and purified using the EZ View Red ANTI-FLAG M2 Affinity Gel (Sigma), according to the manufacturer’s protocol. The purified protein was analyzed by SDS-PAGE and Western blotting (**Supplementary Figures 1d, e**).

### ATP hydrolysis assay of isolated IMVs

ATPase activity of isolated IMVs was measured using the MitoCheck Complex V Activity Assay Kit (Cayman Chemical) according to the manufacturer’s protocol. The ATP hydrolysis rate was calculated from the slope of the absorbance change at 340 nm. The slope was obtained by fitting the change in absorbance with a simple linear regression function in GraphPad Prism (10.1.0). Oligomycin (5 µg/µl) was used as a positive control.

### Cryo-EM grid preparation

Cryo-EM grids (QuantiFoil R 2/1 Au 200 or Cu 300 mesh grid covered with 2 nm carbon; Electron Microscopy Sciences) were either plasma cleaned (0.3 Torr for 15 s at medium settings; Harrick Plasma) or glow discharged (under vacuum at 20 mA for 15 s; EmiTech). 3 μl of prepared IMVs were applied onto the prepared grid and vitrified in liquid ethane at 100% humidity using a Mark IV Vitrobot (Thermo Fisher). The excess solution was removed by blotting for 3-5 s before plunging into liquid ethane. Vitrified grids were immediately stored in liquid N_2_ until data collection.

### Cryo-EM data collection

Cryo-EM data were collected using a Titan Krios G2 microscope (Thermo Fisher Scientific) operating at 300 kV. The microscope was equipped with a post-GIF K3 Summit direct electron detector (TFS) and a BioQuantum energy filter with a slit width of 20 eV. Data collection was automated using the Thermo Fisher EPU software package. Movies were recorded at a nominal magnification of 64,000. The defocus values ranged from -1.0 to -3.0 μm.

### Electrophysiology recordings of ion channels

**Figure 2:**
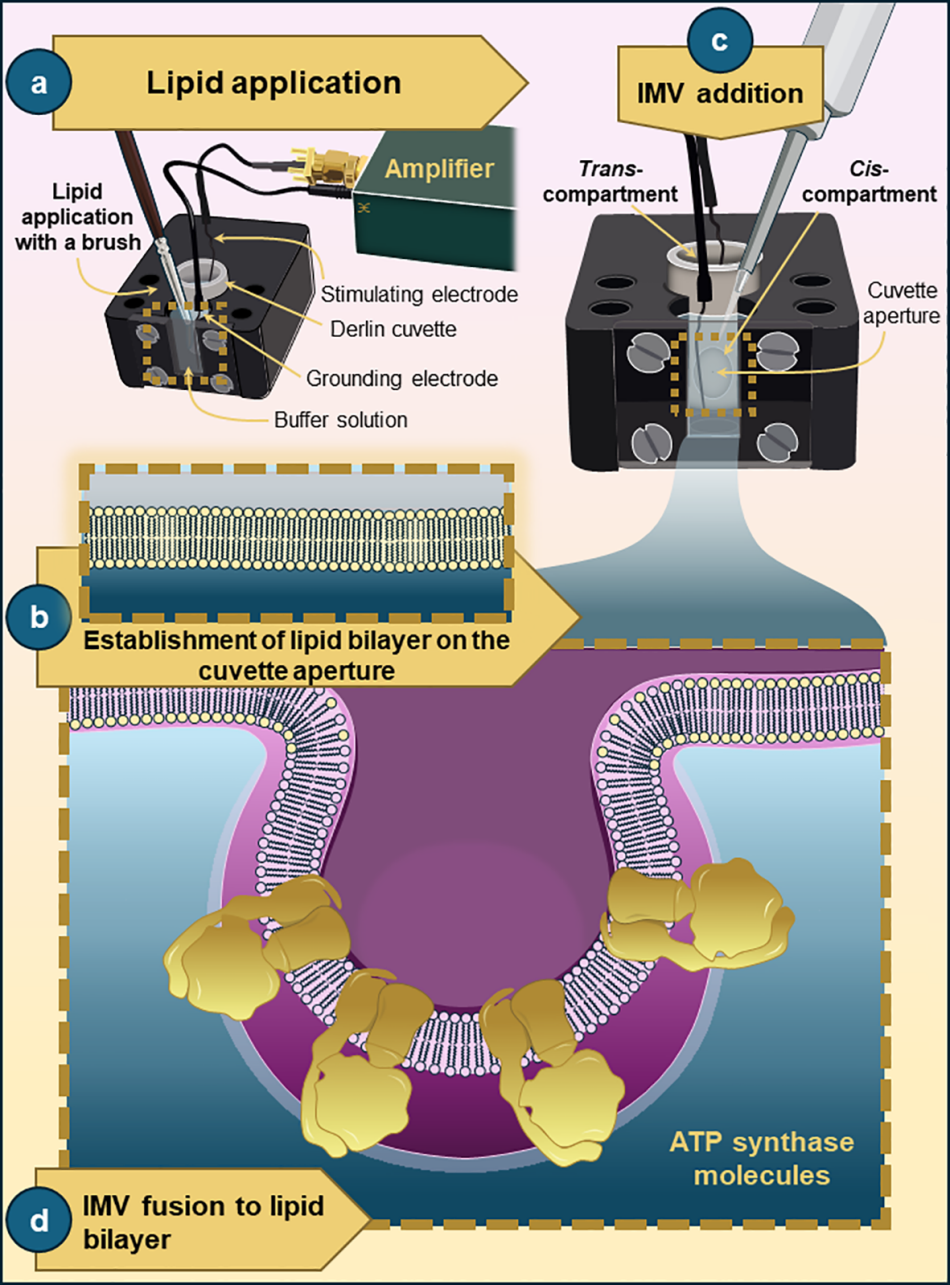
Schematic representation of a planar lipid bilayer experiment. The experiments are performed in a recording chamber with a Derlin cuvette that has two compartments, *cis* and *trans*. These compartments are connected via a small aperture with a diameter of 50-250 μm. **(a)** The lipid mixture, dissolved in decane, is applied to the aperture using a fine paintbrush. **(b, c)** Upon subsequent formation of a planar lipid bilayer membrane on a cuvette aperture, IMVs or purified protein are added to the *cis-*compartment of the chamber. **(d)** The reconstitution of the added IMVs into the bilayer membrane is achieved by diffusion-led vesicle fusion under the influence of applied voltage or short voltage pulses.

**Figure 2** shows a schematic representation of a planar lipid bilayer experiment, including the formation of an artificial lipid bilayer at the cuvette aperture and its subsequent fusion with IMV.

Planar lipid bilayer recordings were performed in a 13 mm Derlin cuvette (Warner Instruments) with a 200 µm aperture (**Figure 2a**). The lipid mixture was prepared by drying an aliquot of L-α-phosphatidylcholine (Avanti Polar Lipids; 20 mg/ml in chloroform) under N_2_ gas in a glass cuvette, then redissolving it in n-decane (Thermo Scientific). After adding intracellular solution (120 mM KCl; 8 mM NaCl; 0.5 mM EGTA; 10 mM HEPES; pH 7.3) to both the *cis* and *trans* compartments of the cuvette, the prepared lipid was directly painted onto the cuvette aperture using a fine paintbrush (**Figures 2a, b**). The stability and goodness of the bilayer were determined by the resistance (5–7 GΩ) and C-pipette values (50–120 pF). 3-10 μl of IMVs were added to the *cis* side of the cuvette. The insertion of the added IMVs into the planar lipid bilayer (**Figure 2c**) was achieved by diffusion-led vesicle fusion under the influence of applied voltage (ranging from -100 to +100 mV). The applied voltage was held constant for 2–5 min, after which it was increased in 10 mV increments. Typically, a constant voltage of +40 mV was applied first, and it was then increased to +60 mV or higher if no channel activity was observed. Similarly, negative-voltage applications were used to achieve successful vesicle fusion and channel activity. Alternatively, short pulses (5–10 s) of varying voltages were manually applied to enhance the frequency of IMV insertion (**Figure 2d**). In cases where no IMV fusion or channel activity was recorded across several trials of voltage application or voltage pulsing, the planar lipid bilayer was disrupted (zapped) and reformed. Upon detection of channel activity, its electrophysiological behavior and pharmacological profile were examined to determine its molecular identity. Final concentrations of 1 mM Ca^2+^, 1 mM ATP, 10 μM BKA, or 2 μM BDQ were added on the *cis* side during recordings to assess their effects on mitochondrial ion channel activity. Data acquisition was performed with an ePatch amplifier (Elements) and EZ Patch Software.

An individual electrophysiology trace was binned to create an all-points histogram of point count as a function of current amplitude. A major peak occurred at the baseline, and small peaks occurred at the current amplitude corresponding to each open channel level. The recorded electrophysiology trace was then fitted for event detection using a single-channel search after selecting discrete open channel current levels (based on the histogram). Each opening and closing event was identified, and the frequency of channel openings (NPo, where N is the number of channels and Po is the open-channel probability) was determined from single-channel event statistics. For data analysis, Clampfit software (Molecular Devices) was used. The measured current was adjusted for the holding voltage, assuming a linear Current-Voltage relationship. The ionic current (*I*) through a typical ion channel is measured in pico-Amperes (pA), and potential or voltage (*V*) is measured in millivolts (mV). The conductance (G) of ions is commonly measured in Siemens (S), with single-channel conductance expressed in nanoSiemens (nS) or picoSiemens (pS), according to the equation G = I/V, where I is in pA, and *V* is in mV. Group data were evaluated in terms of peak conductance and frequency of channel opening, and the error bars represent ± SEM.

## Results

### Single-channel recordings of ACLC using planar lipid bilayer electrophysiology of IMVs

In this study, we developed a robust electrophysiological approach to characterize mitochondrial membrane proteins that play key roles in mPTP. Well-established pharmacological modulators of mitochondrial ion channels were used to identify the recorded channels and depict their functional properties. Porcine heart mitochondria were used for preparing IMVs enriched for ATP synthase (**Figure 1**; **Supplementary Figure S1a**). **Supplementary Figure 1a** shows a representative cryo-EM image of isolated IMVs. White arrowheads show the F_1_ domain of ATP synthase protruding from the membrane. The functional activity of ATP synthase in isolated IMVs was assessed by measuring ATP hydrolysis rate. IMVs exhibited ATP hydrolysis activity inhibited by oligomycin, confirming the presence of a fully coupled and functional ATP synthase in the isolated membranes (**Supplementary Figures 1b, c**). In addition, we purified ATP synthase from porcine heart mitochondria and performed planar lipid bilayer recordings to compare its biophysical properties with those of IMVs.

**Figure 3:**
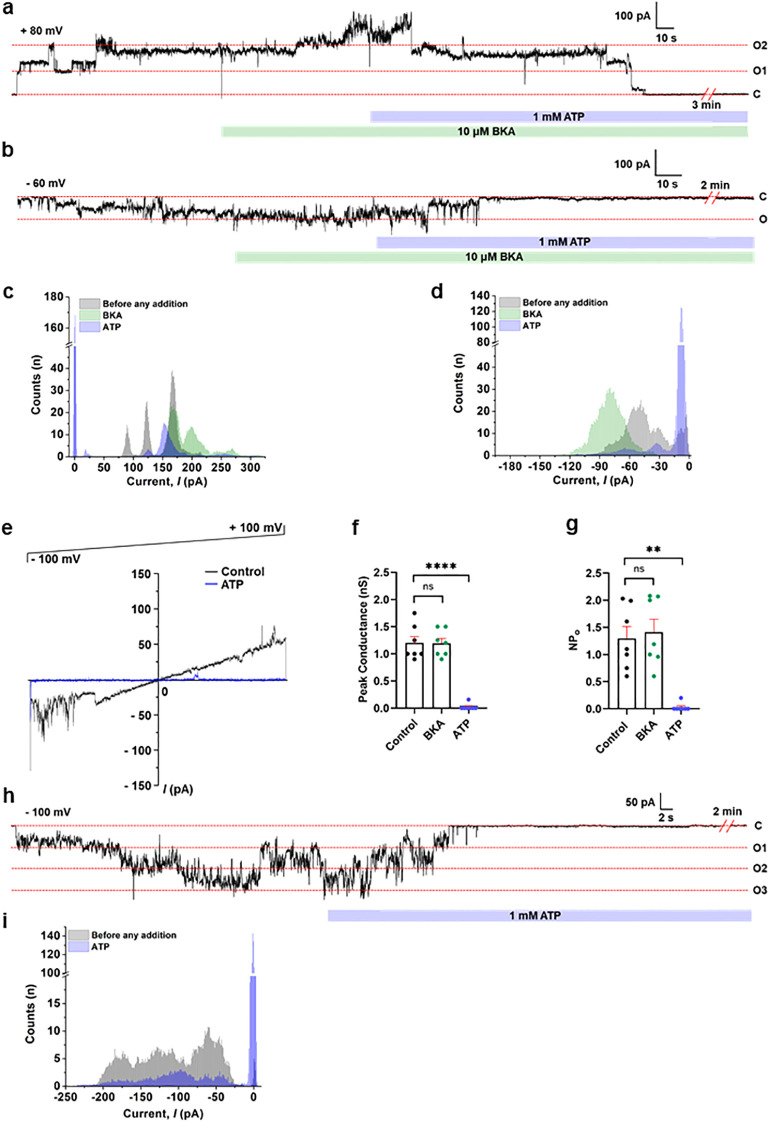
Single-channel recording of ACLC using porcine heart IMVs and purified ATP synthase showing channel inhibition with ATP. **(a, b)** Representative continuous single-channel planar lipid bilayer recordings of IMVs at +80 mV and -60 mV, respectively. The green bar represents the addition of a specific ANT inhibitor, Bongkrekic acid (BKA, 10 μM). The purple bar represents the addition of ATP (1 mM). The trace shown in **(a)** was recorded in the presence of 1 mM Ca^2+^. **(c, d)** The histogram plots show the change in channel current in the presence of BKA, before and after ATP addition for the traces shown in **(a, b)**, respectively. **(e)** Representative voltage ramp showing channel activity in the presence of BKA, before and after ATP (1 mM) addition. **(f)** Group data for the peak channel conductance in the absence and presence of BKA (10 μM) and ATP (1 mM). *P* = 0.8896 for BKA and ****P < 0.0001 for ATP (n=7). Channels were recorded at holding voltages ranging from -100 mV to +100 mV. **(g)** Group data for the frequency of channel opening (NPo) in the absence and presence of BKA (10 μM) and ATP (1 mM). *P* = 0.1227 for BKA and **P < 0.0016 for ATP (n=7). Channels were recorded at holding voltages ranging from -100 mV to +100 mV. **(h)** Representative continuous single-channel planar lipid bilayer recording of purified ATP synthase in the presence of BKA at -100 mV, showing the channel inhibition with ATP (1 mM). **(i)** The histogram plot displays the change in channel current before and after ATP addition, for the trace shown in **(h)**. ‘C’ and ‘O’ refer to the channel’s closed and open state, respectively. Signals were filtered at 5 kHz using the amplifier circuitry. The error bars in panels **(f, g)** are represented as standard error mean (SEM), and a paired t-test was used for statistical analysis. Normalized Counts (n) are represented for all the histograms.

**Figure 3** demonstrates single-channel recordings of ACLC using porcine heart IMVs and purified ATP synthase. **Figure 3a** shows a continuous single-channel recording of IMV performed at +80 mV in the presence of Ca^2+^. The channel was completely inhibited by ATP, but was not sensitive to the ANT inhibitor, BKA (Brustovetsky et al., 2002). Similarly, **Figure 3b** shows a continuous single-channel recording of voltage-gated channel activity at -60 mV that was insensitive to BKA but inhibited by ATP. The histograms show the change in channel current in the presence of BKA, before and after ATP addition (**Figures 3c, d**), for the traces shown in **Figures 3a, b**, respectively. **Figure 3e** shows a representative voltage ramp recording of IMV from -100 mV to +100 mV in the presence of BKA, before and after ATP addition. The addition of ATP inhibited channel activity at all voltages during continuous IMV recordings (**Figure 3e**). The group data for peak conductance and the NPo in the absence and presence of BKA and ATP are shown in **Figures 3f, g**, respectively. The group data for the peak conductance of seven recorded channels in the presence of BKA showed an average conductance of ~1.2 nS, fully inhibited by ATP (**Figure 3f**). **Figure 3h** shows a representative continuous single-channel planar lipid bilayer recording of purified porcine heart ATP synthase in the presence of BKA at -100 mV, demonstrating the inhibition of the channel by ATP. The histograms in **Figure 3i** display the change in channel current before and after ATP addition for the trace shown in **Figure 3h**. A similar channel, with 1.5 nS peak conductance and sensitivity to ATP but not BKA, was reported to form by purified porcine and bovine heart ATP synthase, analysed by patch-clamp recordings of reconstituted proteoliposomes (Alavian et al., 2014; Mnatsakanyan et al., 2019) and the planar lipid bilayer system (Urbani et al., 2019; Kumar et al., 2025). These observations allowed us to suggest that the recorded channel in IMVs (**Figures 3a–g**) is ACLC. 

**Figure 4:**
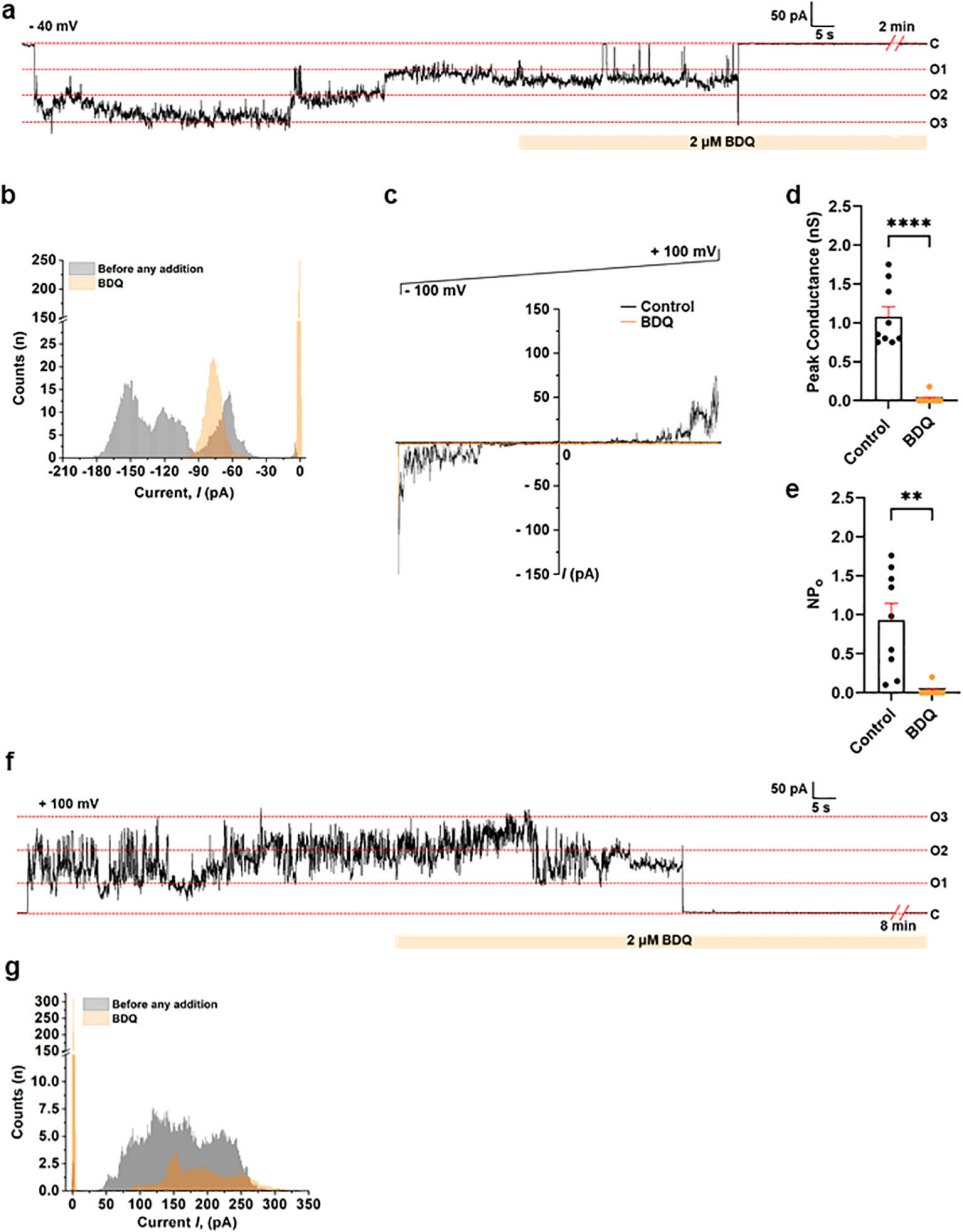
Single-channel recording of ACLC using porcine heart IMVs and purified ATP synthase showing channel inhibition with BDQ. **(a)** Representative continuous single-channel planar lipid bilayer recording of IMVs at -40 mV. The orange bar below the trace represents the addition of a specific ACLC inhibitor, Bedaquiline (BDQ, 2 μM). BKA (10 μM) was present in the cuvette throughout the recording. **(b)** The histogram plot of the trace shown in **(a)** demonstrates a change in channel current before and after the addition of BDQ. **(c)** Representative voltage ramp recording showing channel activity before and after the addition of BDQ (2 μM). Channels were recorded at holding voltages ranging from -100 mV to +100 mV. **(d)** Group data for the peak channel conductance in the absence and presence of BDQ (2 μM). ****P < 0.0001 (n=9). **(e)** Group data for the frequency of channel opening (NPo) in the absence and presence of BDQ (2 μM). **P = 0.0038 (n=9). **(f)** Representative continuous single-channel planar lipid bilayer recording of purified porcine heart ATP synthase at +100 mV, showing the channel inhibition with BDQ (2 μM). **(g)** The histogram plot displays the change in channel current before and after BDQ addition, for the trace shown in **(f)**. ‘C’ and ‘O’ refer to the channel’s closed and open state, respectively. Signals were filtered at 5 kHz using the amplifier circuitry. The error bars in panels **(d, e)** are represented as standard error mean (SEM), and a paired t-test was used for statistical analysis. Normalized Counts (n) are represented for all the histograms.

We further confirmed the identity of BKA-insensitive channel(s) using BDQ, a specific ACLC inhibitor. **Figure 4** depicts a single-channel recording of porcine heart IMVs and purified ATP synthase, which are fully inhibited by BDQ. **Figure 4a** shows a representative continuous single-channel planar lipid bilayer recording of IMVs in the presence of BKA at -40 mV. The addition of a BDQ fully inhibited the channel. The histogram of the trace shown in **Figure 4a** demonstrates a change in channel current before and after the addition of BDQ (**Figure 4b**). BDQ also inhibited IMV channel activity at all voltages (-100 to +100 mV) during voltage-ramp recording (**Figure 4c**). The group data for peak conductance and the NPo in the absence and presence of BDQ are shown in **Figures 4d, e**, respectively. Group data on peak conductance from nine IMV recordings showed an average of ~1.1 nS, which was fully inhibited by BDQ. Similar observations were made in the single-channel recordings of purified porcine heart mitochondrial ATP synthase shown in **Figures 4f, g**, and in our recent study (Kumar et al., 2026). Representative continuous single-channel planar lipid bilayer recording of purified ATP synthase at -100 mV shows that full channel closure occurs upon BDQ addition (**Figure 4f**). The histogram shows the change in channel current before and after BDQ addition for the trace shown in **Figure 4f**. These data suggest that BDQ can be used in IMV recordings to identify ACLC activity.

### Single-channel recordings of ANT using planar lipid bilayer electrophysiology of IMVs

ANT is a highly abundant mitochondrial inner membrane protein that forms a voltage-gated, large-conductance channel and is proposed to play a role in mPTP (Brustovetsky and Klingenberg, 1996; Brustovetsky et al., 2002; Halestrap and Brenner, 2003). Thus, next, we focused on recording and characterizing channels sensitive to the ANT inhibitor, BKA, in planar lipid bilayer recordings of IMVs. We also used purified ANT in planar bilayer recordings for comparative channel analysis (**Supplementary Figures 1d, e**).

**Figure 5:**
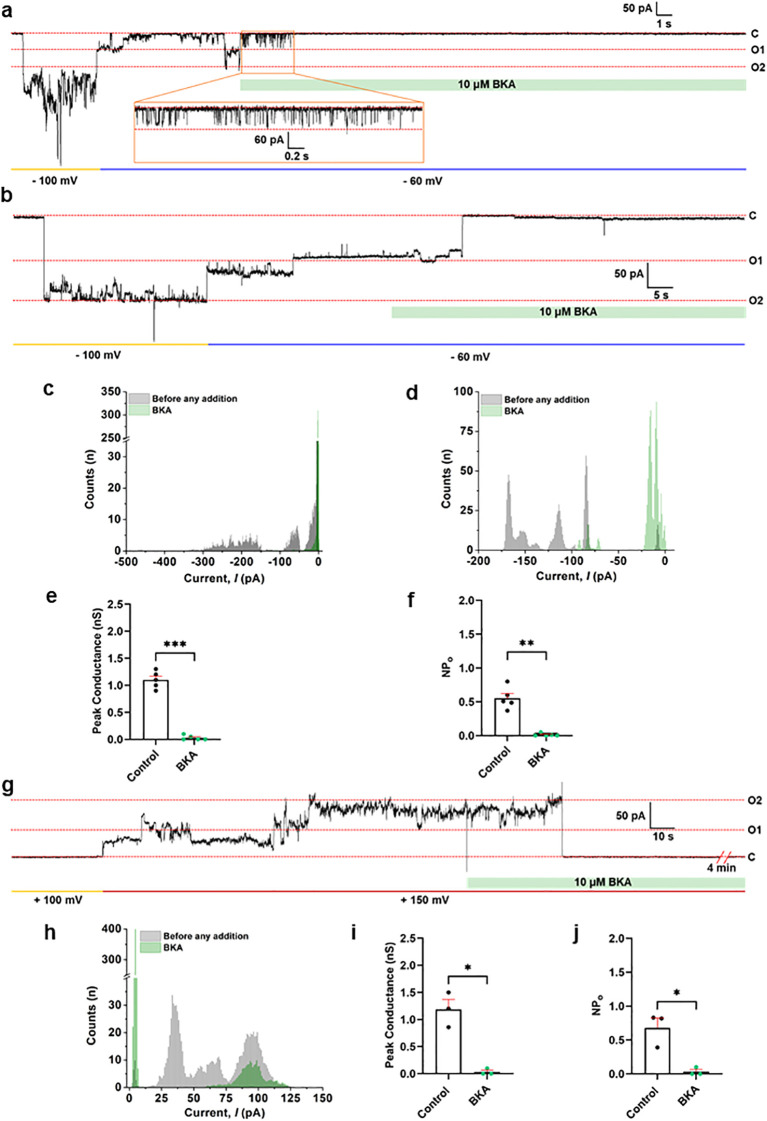
Single-channel recording of the ANT channel using porcine heart IMVs and purified ANT showing channel inhibition with BKA. **(a, b)** Representative continuous single-channel planar lipid bilayer recording of IMVs performed initially at -100 mV and then at -60 mV. The green bar in panels **(a, b)** indicates the addition of a specific ANT channel inhibitor, Bongkrekick acid (BKA). **(c, d)** The histogram plots show the change in channel current before and after BKA, for traces shown in **(a, b)**, respectively. **(e)** Group data for the peak channel conductance in the absence and presence of BKA (10 μM). ***P = 0.0002, (n=5). **(f)** Group data for the frequency of channel opening (NPo) in the absence and presence of BKA (10 μM). **P = 0.0019 (n=5). **(g)** A continuous single-channel planar lipid bilayer recording of purified murine ANT at +150 mV, showing the channel inhibition with BKA (10 μM). **(h)** Histogram plot showing the change in channel current before and after BKA, for the trace shown in **(g)**. **(i)** Group data for the peak channel conductance in the absence and presence of BKA (10 μM) for channels obtained with purified ANT. *P = 0.0330, (n=3). **(j)** Group data for the frequency of channel opening (NPo) in the absence and presence of BKA (10 μM) for channels obtained with purified ANT. *P = 0.0392 (n=3). ‘C’ and ‘O’ refer to the channel’s closed and open state, respectively. Signals were filtered at 5 kHz using the amplifier circuitry. The error bars in panels **(e, f, i, j)** are represented as standard error mean (SEM), and a paired t-test was used for statistical analysis. Normalized Counts (n) are represented for all the histograms.

**Figure 5** demonstrates the single-channel recording of IMVs and purified ANT protein. **Figures 5a, b** show continuous single-channel recordings of a voltage-gated channel. A holding voltage of -100 mV induces a channel with a peak conductance of ~2.5 nS, which subsides to ~1.2 nS at -60 mV in both traces (**Figures 5a, b**). The recorded channel activity was inhibited by the addition of BKA (**Figures 5a, b**). The histogram plots show the change in channel current before and after BKA (**Figures 5c, d**), for the traces shown in **Figures 5a, b**, respectively. The group data for peak conductance and the NPo in the absence and presence of BKA are shown in **Figures 5e, f**, respectively. The average peak conductance for this subset of BKA-inhibited channels was ~1.1 nS (**Figure 5e**). Notably, these channels exhibited a distinct pharmacological profile compared with those shown in **Figure 3** and **4**, which were not sensitive to BKA but were fully inhibited by ATP. In this case, BKA alone inhibited the channel (**Figure 5**). These findings suggest that the ANT was the channel recorded in IMV recordings.

Next, we performed single-channel planar lipid bilayer recordings of purified ANT for comparative functional analysis. We show that purified ANT forms a voltage-gated channel sensitive to BKA and ATP (**Figures 5g–j**; **Supplementary Figure S2**), as reported earlier (Brustovetsky and Klingenberg, 1996). **Figure 5g** demonstrates a continuous single-channel planar lipid bilayer recording of purified ANT at +150 mV, which is inhibited upon addition of BKA. Histogram plots (**Figure 5h**) show the change in channel current before and after BKA addition for the trace shown in **Figure 5g**. **Figures 5i, j** show the group data for peak conductance and NPo, respectively, for channels obtained from purified ANT. The average peak conductance obtained for purified ANT channels was 1.18 nS (**Figure 5i**), and it was markedly inhibited by BKA (**Figures 5i, j**). **Supplementary Figure 2** shows truncated traces from continuous single-channel planar lipid bilayer recordings of purified ANT at a holding voltage of -100 mV and +100 mV. Traces show a decrease in channel peak current with increasing BKA concentration at +100 mV. Channel activity was completely inhibited with ATP. The histograms for each corresponding trace show a gradual decrease in channel current upon the addition of BKA and ATP. A similar peak conductance, probability of channel opening, and pharmacological inhibition profile of purified ANT channels suggest that the recorded channels using IMVs in **Figures 5a, b** can be assigned to ANT.

In addition to channels sensitive to Ca^2+^, BKA, BDQ, or ATP, other channels insensitive to these agents were also randomly and infrequently observed during IMV recordings. Thus, planar lipid bilayer recordings of IMVs can also serve as an exploratory platform for identifying and analyzing novel, unexplored channels of the mitochondrial inner membrane.

## Discussion

In this study, we examined mitochondrial inner membrane ion conductance by planar lipid bilayer recordings of IMVs enriched with ATP synthase. We used various pharmacological agents that specifically modulate the channel conductance of ATP synthase and ANT to dissect and analyse the electrophysiological properties of these channels.

A prevalent subset of channels recorded from IMVs exhibited high-conductance (~1.1 nS) activity that was insensitive to BKA but fully inhibited by ATP and BDQ, a specific inhibitor of ACLC. The pharmacological profile and single-channel properties of this subset of recorded channels closely resemble those of the mammalian ACLC, as described in current and past studies (Alavian et al., 2014; Mnatsakanyan et al., 2019; Urbani et al., 2019). The channel formed by purified porcine heart ATP synthase exhibited large-conductance voltage-gated activity and multiple subconductance states. The addition of Ca^2+^ further increased the frequency of channel opening, whereas other divalent and trivalent ions (Ba^2+^, Gd^3+^), ATP, and BDQ inhibited its activity, as described before (Mnatsakanyan et al., 2019; Kumar et al., 2026).

A second subset of channels recorded in lipid bilayer recordings of IMVs still demonstrated high conductance (~1.0 nS) and voltage-gated activity but were sensitive to BKA, thus closely resembling the channel properties attributed to ANT. In this study, we found that murine ANT, expressed and purified from HEK293 cells, forms channels with peak conductance and opening frequency similar to those observed in IMV recordings, and these channels are inhibited by BKA and ATP. The recombinant ANT from *Neurospora crassa*, expressed and purified from *E. coli* (Brustovetsky et al., 2002), formed a channel with a conductance of 500–700 pS in the buffer with the composition of 100 mM KCl, 2 mM MgCl_2_, 1 mM CaCl_2_, 4 mM K-gluconate, 5 mM MES, 5 mM Tris at pH 7.4. The channel was inhibited by BKA and completely blocked by BKA and ADP in patch-clamp studies.

Collectively, these findings demonstrate that reconstitution of inner mitochondrial membrane vesicles into planar lipid bilayers provides a powerful and versatile platform for studying both well-characterized and yet-to-be-identified voltage- or ligand-gated ion channels under controlled experimental conditions. This approach bridges the gap between planar lipid bilayer studies of purified, reconstituted proteins and patch-clamp recordings of mitochondrial membranes, offering several advantages. One advantage of this methodology is that it permits the straightforward application of pharmacological agents to both the *cis* and *trans* compartments of the cuvette during recordings, facilitating the evaluation of their modulatory effects on ion conductance.

Additionally, this approach can be extended to native vesicles derived from other cellular organelles, enabling detailed investigation of ion channel gating and regulatory mechanisms within their native lipid environments at single-channel resolution.

## Data Availability

The original contributions presented in the study are included in the article/**Supplementary Material**. Further inquiries can be directed to the corresponding author.

## Supplementary material

Supplementary Figure 1 Isolation of mitochondrial IMVs and purification of ANT. **(a)**. Representative cryo-electron microscopy image of IMVs. White arrowheads represent the hydrophilic F_1_ domain of ATP synthase that protrudes out from the membrane. Scale bar is 200 nm. **(b)**. ATP hydrolysis assay of IMVs. A representative change in absorbance at 340 nm, reflecting the rate of hydrolysis. **(c)**. Group data of ATPase activity of IMVs. Oligomycin (5 µg/µL) was used as a positive control (n=6). The assay was performed with 3.24 µg of IMVs isolated from porcine heart mitochondria. Error bars are represented as SEM. The unpaired t-test is used for statistical analysis. *****P* < 0.0001. **(d, e)**. Silver stain and Western blot analysis of purified ANT, respectively.

Supplementary Figure 2 Planar lipid bilayer electrophysiology recording of purified ANT. Truncated traces from continuous single-channel planar lipid bilayer recording of purified murine ANT at a holding potential of -100 mV and +100 mV. Traces show a decrease in channel current with increasing BKA concentration at +100 mV. Channel activity is completely inhibited with ATP (1 mM). Histograms shown on the right panel for each corresponding trace demonstrate a gradual decrease in channel current upon addition of BKA and ATP. ‘C’ and ‘O’ refer to the channel’s closed and open state, respectively. Signals were filtered at 5 kHz using the amplifier circuitry.

## References

[B57] Alavian K. N. Beutner G. Lazrove E. Sacchetti S. Park H. A. Licznerski P. . (2014). An uncoupling channel within the c-subunit ring of the F1FO ATP synthase is the mitochondrial permeability transition pore. Proc. Natl. Acad. Sci. U.S.A. 111, 10580–10585. doi: 10.1073/pnas.1401591111. PMID: 24979777 PMC4115574

[B56] Azarashvili T. Odinokova I. Bakunts A. Ternovsky V. Krestinina O. Tyynelä J. . (2014). Potential role of subunit c of F0F1-ATPase and subunit c of storage body in the mitochondrial permeability transition. Effect of the phosphorylation status of subunit c on pore opening. Cell. Calcium 55, 69–77. doi: 10.1016/j.ceca.2013.12.002. PMID: 24380588

[B3] Bernardi P. Gerle C. Halestrap A. P. Jonas E. A. Karch J. Mnatsakanyan N. . (2023). Identity, structure, and function of the mitochondrial permeability transition pore: controversies, consensus, recent advances, and future directions. Cell Death Differ. 30, 1869–1885. doi: 10.1038/s41418-023-01187-0. PMID: 37460667 PMC10406888

[B33] Bertholet A. M. (2021). The use of the patch-clamp technique to study the thermogenic capacity of mitochondria. J. Vis. Exp. 171. doi: 10.1016/j.bbabio.2024.149181. PMID: 33999016

[B13] Bertholet A. M. Natale A. M. Bisignano P. Suzuki J. Fedorenko A. Hamilton J. . (2022). Mitochondrial uncouplers induce proton leak by activating AAC and UCP1. Nature 606, 180–187. doi: 10.1016/j.bpj.2021.11.244. PMID: 35614225 PMC9646675

[B12] Bertholet A. M. Kirichok Y. (2022). Mitochondrial H(+) leak and thermogenesis. Annu. Rev. Physiol. 84, 381–407. doi: 10.1146/annurev-physiol-021119-034405. PMID: 34758268 PMC8976115

[B54] Bonora M. Bononi A. De Marchi E. Giorgi C. Lebiedzinska M. Marchi S. . (2013). Role of the c subunit of the FO ATP synthase in mitochondrial permeability transition. Cell. Cycle 12, 674–683. doi: 10.4161/cc.23599. PMID: 23343770 PMC3594268

[B68] Brustovetsky N. Klingenberg M. (1996). Mitochondrial ADP/ATP carrier can be reversibly converted into a large channel by Ca2+. Biochemistry 35, 8483–8488. doi: 10.1021/bi960833v. PMID: 8679608

[B66] Brustovetsky N. Tropschug M. Heimpel S. Heidkamper D. Klingenberg M. (2002). A large Ca2+-dependent channel formed by recombinant ADP/ATP carrier from Neurospora crassa resembles the mitochondrial permeability transition pore. Biochemistry 41, 11804–11811. doi: 10.1002/9781119017127.ch1. PMID: 12269823

[B49] Carraro M. Giorgio V. Šileikytė J. Sartori G. Forte M. Lippe G. . (2014). Channel formation by yeast F-ATP synthase and the role of dimerization in the mitochondrial permeability transition. J. Biol. Chem. 289, 15980–15985. doi: 10.1016/j.bbabio.2014.05.192. PMID: 24790105 PMC4047373

[B50] Carraro M. Checchetto V. Sartori G. Kucharczyk R. di Rago J. P. Minervini G. . (2018). High-Conductance Channel Formation in Yeast Mitochondria is Mediated by F-ATP Synthase e and g Subunits. Cell. Physiol. Biochem. 50, 1840–1855. doi: 10.1159/000494864. PMID: 30423558

[B70] Chan T. L. Greenawalt J. W. Pedersen P. L. (1970). Biochemical and ultrastructural properties of a mitochondrial inner membrane fraction deficient in outer membrane and matrix activities. J. Cell Biol. 45, 291–305. doi: 10.1083/jcb.45.2.291. PMID: 4254678 PMC2107903

[B5] Colombini M. (2004). VDAC: the channel at the interface between mitochondria and the cytosol. Mol. Cell. Biochem. 256-257, 107–115. doi: 10.1023/b:mcbi.0000009862.17396.8d. PMID: 14977174

[B36] Colombini M. (2007). Measurement of VDAC permeability in intact mitochondria and in reconstituted systems. Methods Cell. Biol. 80, 241–260. doi: 10.1016/s0091-679x(06)80012-9. PMID: 17445698

[B4] Colombini M. Mannella C. A. (2012). VDAC, the early days. Biochim. Biophys. Acta 1818, 1438–1443. doi: 10.1016/j.bbamem.2011.11.014. PMID: 22120576 PMC3296906

[B25] Dunlop J. Bowlby M. Peri R. Vasilyev D. Arias R. (2008). High-throughput electrophysiology: an emerging paradigm for ion-channel screening and physiology. Nat. Rev. Drug Discov. 7, 358–368. doi: 10.1038/nrd2552. PMID: 18356919

[B10] Garlid K. D. Paucek P. (2003). Mitochondrial potassium transport: the K(+) cycle. Biochim. Biophys. Acta 1606, 23–41. doi: 10.1080/15216540152845948. PMID: 14507425

[B48] Giorgio V. von Stockum S. Antoniel M. Fabbro A. Fogolari F. Forte M. . (2013). Dimers of mitochondrial ATP synthase form the permeability transition pore. Proc. Natl. Acad. Sci. U.S.A. 110, 5887–5892. doi: 10.1073/pnas.1217823110. PMID: 23530243 PMC3625323

[B59] Giorgio V. Burchell V. Schiavone M. Bassot C. Minervini G. Petronilli V. . (2017). Ca(2+) binding to F-ATP synthase beta subunit triggers the mitochondrial permeability transition. EMBO Rep. 18, 1065–1076. doi: 10.15252/embr.201643354. PMID: 28507163 PMC5494526

[B62] Guo H. Courbon G. M. Bueler S. A. Mai J. Liu J. Rubinstein J. L. (2021). Structure of mycobacterial ATP synthase bound to the tuberculosis drug bedaquiline. Nature 589, 143–147. doi: 10.1038/s41586-020-3004-3. PMID: 33299175

[B51] Guo L. Carraro M. Carrer A. Minervini G. Urbani A. Masgras I. . (2019). Arg-8 of yeast subunit e contributes to the stability of F-ATP synthase dimers and to the generation of the full-conductance mitochondrial megachannel. J. Biol. Chem. 294, 10987–10997. doi: 10.1074/jbc.ra119.008775. PMID: 31160339 PMC6635451

[B46] Halestrap A. P. Brenner C. (2003). The adenine nucleotide translocase: a central component of the mitochondrial permeability transition pore and key player in cell death. Curr. Med. Chem. 10, 1507–1525. doi: 10.2174/0929867033457278. PMID: 12871123

[B7] Inoue I. Nagase H. Kishi K. Higuti T. (1991). ATP-sensitive K+ channel in the mitochondrial inner membrane. Nature 352, 244–247. doi: 10.1038/352244a0. PMID: 1857420

[B41] Jiang Q. X. Gonen T. (2012). The influence of lipids on voltage-gated ion channels. Curr. Opin. Struct. Biol. 22, 529–536. doi: 10.1016/j.sbi.2012.03.009. PMID: 22483432 PMC3408884

[B30] Jonas E. A. Knox R. J. Kaczmarek L. K. (1997). Giga-ohm seals on intracellular membranes: a technique for studying intracellular ion channels in intact cells. Neuron 19, 7–13. doi: 10.1016/s0896-6273(00)80343-8. PMID: 9247259

[B28] Kinnally K. W. Antonenko Y. N. Zorov D. B. (1992). Modulation of inner mitochondrial membrane channel activity. J. Bioenerg. Biomembr. 24, 99–110. doi: 10.1007/bf00769536. PMID: 1380510

[B27] Kinnally K. W. Campo M. L. Tedeschi H. (1989). Mitochondrial channel activity studied by patch-clamping mitoplasts. J. Bioenerg. Biomembr. 21, 497–506. doi: 10.1007/bf00762521. PMID: 2478535

[B38] Kinnally K. W. Lohret T. A. Campo M. L. Mannella C. A. (1996). Perspectives on the mitochondrial multiple conductance channel. J. Bioenerg. Biomembr. 28, 115–123. doi: 10.1007/bf02110641. PMID: 9132409

[B23] Kinnally K. W. Martinez-Caballero S. Dejean L. M. (2006). Detection of the mitochondrial apoptosis-induced channel (MAC) and its regulation by Bcl-2 family proteins. Curr. Protoc. Toxicol. Chapter 2, Unit2 12. doi: 10.1002/0471140856.tx0212s30. PMID: 20941703

[B22] Kinnally K. W. Zorov D. Antonenko Y. Perini S. (1991). Calcium modulation of mitochondrial inner membrane channel activity. Biochem. Biophys. Res. Commun. 176, 1183–1188. doi: 10.1016/0006-291x(91)90410-9. PMID: 1710110

[B6] Kirichok Y. Krapivinsky G. Clapham D. E. (2004). The mitochondrial calcium uniporter is a highly selective ion channel. Nature 427, 360–364. doi: 10.1038/nature02246. PMID: 14737170

[B67] Kumar A. da Fonseca Rezende e Mello J. Wu Y. Morris D. Mezghani I. Smith E. . (2025). Cryo-EM structure of the brine shrimp mitochondrial ATP synthase suggests an inactivation mechanism for the ATP synthase leak channel. Cell Death Differ. 32, 1518–1535. doi: 10.1038/s41418-025-01476-w. PMID: 40108410 PMC12325954

[B61] Kumar E. S. A. Ikram Mezghani Subhash Eedarapalli Yangyu Wu Khondoker Adeba Ferdous Emma Amjad . (2026). “ Bedaquiline inhibits the ATP synthase leak channel and prevents glutamate-induced neuronal death,” in bioRxiv. 10.1016/j.bpr.2026.100265 PMC13234225 42107841

[B47] Kwong J. Q. Davis J. Baines C. P. Sargent M. A. Karch J. Wang X. . (2014). Genetic deletion of the mitochondrial phosphate carrier desensitizes the mitochondrial permeability transition pore and causes cardiomyopathy. Cell Death Differ. 21, 1209–1217. doi: 10.1038/cdd.2014.36. PMID: 24658400 PMC4085527

[B21] Lohret T. A. Murphy R. C. Drgon T. Kinnally K. W. (1996). Activity of the mitochondrial multiple conductance channel is independent of the adenine nucleotide translocator. J. Biol. Chem. 271, 4846–4849. doi: 10.1074/jbc.271.9.4846. PMID: 8617754

[B64] Luo M. Zhou W. Patel H. Srivastava A. P. Symersky J. Bonar M. M. . (2020). Bedaquiline inhibits the yeast and human mitochondrial ATP synthases. Commun. Biol. 3, 452. doi: 10.1038/s42003-020-01173-z. PMID: 32814813 PMC7438494

[B60] Mahajan R. (2013). Bedaquiline: First FDA-approved tuberculosis drug in 40 years. Int. J. Appl. Basic Med. Res. 3, 1–2. doi: 10.4103/2229-516x.112228. PMID: 23776831 PMC3678673

[B1] Miyawaki T. Mashiko T. Ofengeim D. Flannery R. J. Noh K. M. Fujisawa S. . (2008). Ischemic preconditioning blocks BAD translocation, Bcl-xL cleavage, and large channel activity in mitochondria of postischemic hippocampal neurons. Proc. Natl. Acad. Sci. U.S.A. 105, 4892–4897. doi: 10.1073/pnas.0800628105. PMID: 18347331 PMC2290755

[B32] Mnatsakanyan E. A. J. N. (2017). Examination of mitochondrial ion conductance by patch clamp in intact neurons and mitochondrial membrane preparations. Neuromethods. doi: 10.1007/978-1-4939-6890-9_11. PMID: 41884704

[B17] Mnatsakanyan N. Beutner G. Porter G. A. Alavian K. N. Jonas E. A. (2017). Physiological roles of the mitochondrial permeability transition pore. J. Bioenerg. Biomembr. 49, 13–25. doi: 10.1007/s10863-016-9652-1. PMID: 26868013 PMC4981558

[B58] Mnatsakanyan N. Llaguno M. C. Yang Y. Yan Y. Weber J. Sigworth F. J. . (2019). A mitochondrial megachannel resides in monomeric F1FO ATP synthase. Nat. Commun. 10, 5823. doi: 10.1038/s41467-019-13766-2. PMID: 31862883 PMC6925261

[B35] Mnatsakanyan N. Park H. A. Wu J. He X. Llaguno M. C. Latta M. . (2022). Mitochondrial ATP synthase c-subunit leak channel triggers cell death upon loss of its F(1) subcomplex. Cell Death Differ. 29, 1874–1887. doi: 10.1038/s41418-022-00972-7. PMID: 35322203 PMC9433415

[B55] Morciano G. Preti D. Pedriali G. Aquila G. Missiroli S. Fantinati A. . (2018). Discovery of novel 1,3,8-triazaspiro[4.5]decane derivatives that target the c subunit of F1/FO-adenosine triphosphate (ATP) synthase for the treatment of reperfusion damage in myocardial infarction. J. Med. Chem. 61, 7131–7143. doi: 10.1021/acs.jmedchem.8b00278. PMID: 30060655

[B53] Neginskaya M. A. Solesio M. E. Berezhnaya E. V. Amodeo G. F. Mnatsakanyan N. Jonas E. A. . (2019). ATP synthase C-subunit-deficient mitochondria have a small cyclosporine A-sensitive channel, but lack the permeability transition pore. Cell Rep. 26, 11–17.e12. doi: 10.1016/j.celrep.2018.12.033. PMID: 30605668 PMC6521848

[B34] Neginskaya M. A. Pavlov E. V. (2023). Investigation of properties of the mitochondrial permeability transition pore using whole-mitoplast patch-clamp technique. DNA Cell Biol. 42, 481–487. doi: 10.1089/dna.2023.0171. PMID: 37311169 PMC10460960

[B2] Patron M. Raffaello A. Granatiero V. Tosatto A. Merli G. De Stefani D. . (2013). The mitochondrial calcium uniporter (MCU): molecular identity and physiological roles. J. Biol. Chem. 288, 10750–10758. doi: 10.1074/jbc.r112.420752. PMID: 23400777 PMC3624455

[B31] Pavlov E. Zakharian E. Bladen C. Diao C. T. Grimbly C. Reusch R. N. . (2005). A large, voltage-dependent channel, isolated from mitochondria by water-free chloroform extraction. Biophys. J. 88, 2614–2625. doi: 10.1529/biophysj.104.057281. PMID: 15695627 PMC1305358

[B16] Petronilli V. Miotto G. Canton M. Brini M. Colonna R. Bernardi P. . (1999). Transient and long-lasting openings of the mitochondrial permeability transition pore can be monitored directly in intact cells by changes in mitochondrial calcein fluorescence. Biophys. J. 76, 725–734. doi: 10.1016/s0006-3495(99)77239-5. PMID: 9929477 PMC1300077

[B18] Petronilli V. Szabo I. Zoratti M. (1989). The inner mitochondrial membrane contains ion-conducting channels similar to those found in bacteria. FEBS Lett. 259, 137–143. doi: 10.1016/0014-5793(89)81513-3. PMID: 2480918

[B15] Ponnalagu D. Gururaja Rao S. Farber J. Xin W. Hussain A. T. Shah K. . (2016). Molecular identity of cardiac mitochondrial chloride intracellular channel proteins. Mitochondrion 27, 6–14. doi: 10.1016/j.mito.2016.01.001. PMID: 26777142

[B14] Ponnalagu D. Singh H. (2017). Anion channels of mitochondria. Handb. Exp. Pharmacol. 240, 71–101. doi: 10.1007/164_2016_39. PMID: 27783269 PMC5855116

[B63] Preiss L. Langer J. D. Yildiz Ö. Eckhardt-Strelau L. Guillemont J. E. Koul A. . (2015). Structure of the mycobacterial ATP synthase Fo rotor ring in complex with the anti-TB drug bedaquiline. Sci. Adv. 1, e1500106. doi: 10.1126/sciadv.1500106. PMID: 26601184 PMC4640650

[B24] Priest B. T. Swensen A. M. McManus O. B. (2007). Automated electrophysiology in drug discovery. Curr. Pharm. Des. 13, 2325–2337. doi: 10.2174/138161207781368701. PMID: 17692004

[B37] Rosencrans W. M. Rajendran M. Bezrukov S. M. Rostovtseva T. K. (2021). VDAC regulation of mitochondrial calcium flux: From channel biophysics to disease. Cell. Calcium 94, 102356. doi: 10.1016/j.ceca.2021.102356. PMID: 33529977 PMC7914209

[B69] Sacchetti S. Alavian K. N. Lazrove E. Jonas E. A. (2013). F1FO ATPase vesicle preparation and technique for performing patch clamp recordings of submitochondrial vesicle membranes. J. Vis. Exp. 75, e4394. doi: 10.3791/4394-v. PMID: 23685483 PMC3676267

[B11] Sanghvi S. Szteyn K. Ponnalagu D. Sridharan D. Lam A. Hansra I. . (2022). Inhibition of BK(Ca) channels protects neonatal hearts against myocardial ischemia and reperfusion injury. Cell. Death Discov. 8, 175. doi: 10.1038/s41420-022-00980-z. PMID: 35393410 PMC8989942

[B42] Saponaro A. Lolicato M. (2022). Editorial: The key role of lipids in the regulation of ion channels. Front. Physiol. 13. doi: 10.3389/fphys.2022.1000082. PMID: 36160863 PMC9490410

[B43] Szabo I. Bernardi P. Zoratti M. (1992). Modulation of the mitochondrial megachannel by divalent cations and protons. J. Biol. Chem. 267, 2940–2946 1371109

[B45] Szabo I. De Pinto V. Zoratti M. (1993). The mitochondrial permeability transition pore may comprise VDAC molecules. II. The electrophysiological properties of VDAC are compatible with those of the mitochondrial megachannel. FEBS Lett. 330, 206–210. doi: 10.1016/0014-5793(93)80274-x, PMID: 7689984

[B8] Szabo I. Szewczyk A. (2023). Mitochondrial ion channels. Annu. Rev. Biophys. 52, 229–254. doi: 10.1146/annurev-biophys-092622-094853. PMID: 37159294

[B19] Szabo I. Zoratti M. (1992). The mitochondrial megachannel is the permeability transition pore. J. Bioenerg. Biomembr. 24, 111–117. doi: 10.1007/BF00769537, PMID: 1380498

[B44] Szabo I. Zoratti M. (1993). The mitochondrial permeability transition pore may comprise VDAC molecules. I. Binary structure and voltage dependence of the pore. FEBS Lett. 330, 201–205. doi: 10.1016/0014-5793(93)80273-w, PMID: 7689983

[B26] Szabo I. Zoratti M. (2014). Mitochondrial channels: ion fluxes and more. Physiol. Rev. 94, 519–608. doi: 10.1152/physrev.00021.2013. PMID: 24692355

[B9] Szewczyk A. Bednarczyk P. Kulawiak B. Żochowska M. Kalenik B. Lewandowska J. . (2025). Mitochondrial potassium channels: New properties and functions. Biochim. Biophys. Acta Bioenerg. 1866, 149546. doi: 10.1016/j.bbabio.2024.149192. PMID: 39933686

[B52] Urbani A. Giorgio V. Carrer A. Franchin C. Arrigoni G. Jiko C. . (2019). Purified F-ATP synthase forms a Ca(2+)-dependent high-conductance channel matching the mitochondrial permeability transition pore. Nat. Commun. 10, 4341. doi: 10.1038/s41467-019-12331-1. PMID: 31554800 PMC6761146

[B40] Zakharian E. (2013). Recording of ion channel activity in planar lipid bilayer experiments. Methods Mol. Biol. 998, 109–118. doi: 10.1007/978-1-62703-351-0_8. PMID: 23529424 PMC3644985

[B39] Zakharian E. (2021). Ion channel reconstitution in lipid bilayers. Methods Enzymol. 652, 273–291. doi: 10.1007/978-1-4939-9446-5_10. PMID: 34059285

[B65] Zhang Y. Lai Y. Zhou S. Ran T. Zhang Y. Zhao Z. . (2024). Inhibition of M. tuberculosis and human ATP synthase by BDQ and TBAJ-587. Nature 631, 409–414. doi: 10.1038/s41586-024-07605-8. PMID: 38961288

[B20] Zoratti M. Szabo I. (1995). The mitochondrial permeability transition. Biochim. Biophys. Acta 1241, 139–176. doi: 10.1016/0304-4157(95)00003-a. PMID: 7640294

[B29] Zorov D. B. Kinnally K. W. Perini S. Tedeschi H. (1992). Multiple conductance levels in rat heart inner mitochondrial membranes studied by patch clamping. Biochim. Biophys. Acta 1105, 263–270. doi: 10.1016/0005-2736(92)90203-x, PMID: 1586662

