# Supplementary figures and images for: Investigation of mitochondrial inner membrane ion conductance by planar lipid bilayer electrophysiology
Source: Front Physiol. 2026 Apr 20;17:1782998. doi: 10.3389/fphys.2026.1782998 (PMC13137367; doi:10.3389/fphys.2026.1782998)

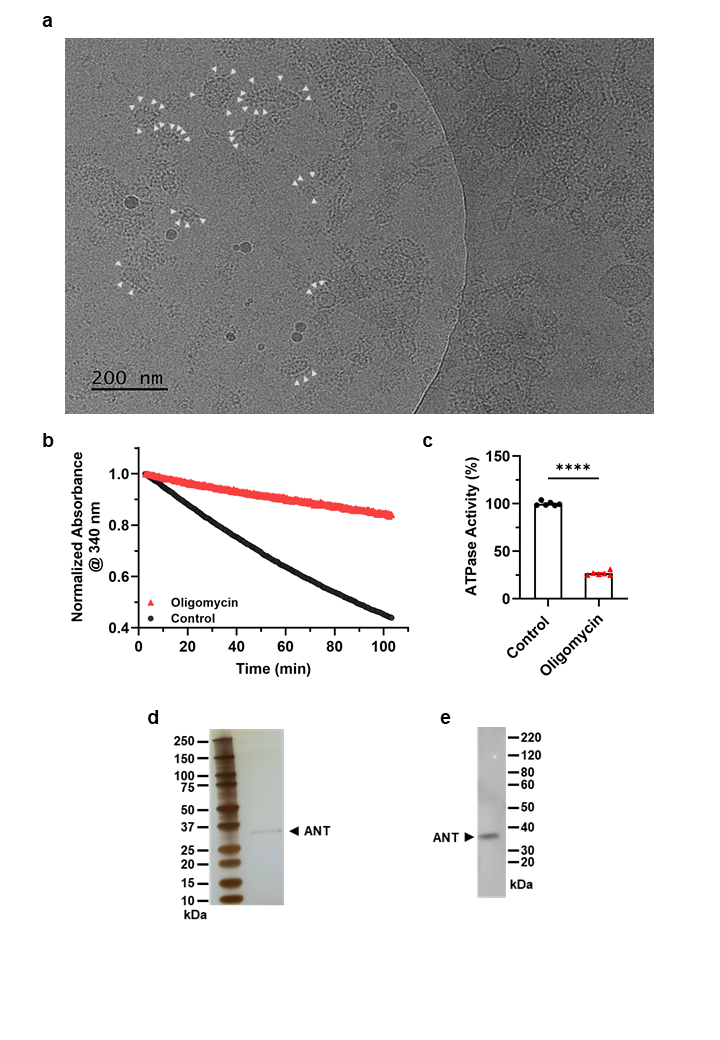

Supplement: Supplementary Figure 1 — Isolation of mitochondrial IMVs and purification of ANT. (a). Representative cryo-electron microscopy image of IMVs. White arrowheads represent the hydrophilic F1 domain of ATP synthase that protrudes out from the membrane. Scale bar is 200 nm. (b). ATP hydrolysis assay of IMVs. A representative change in absorbance at 340 nm, reflecting the rate of hydrolysis. (c). Group data of ATPase activity of IMVs. Oligomycin (5 µg/µL) was used as a positive control (n=6). The assay was performed with 3.24 µg of IMVs isolated from porcine heart mitochondria. Error bars are represented as SEM. The unpaired t-test is used for statistical analysis. ****P < 0.0001. (d, e). Silver stain and Western blot analysis of purified ANT, respectively. [file Image1.tif]

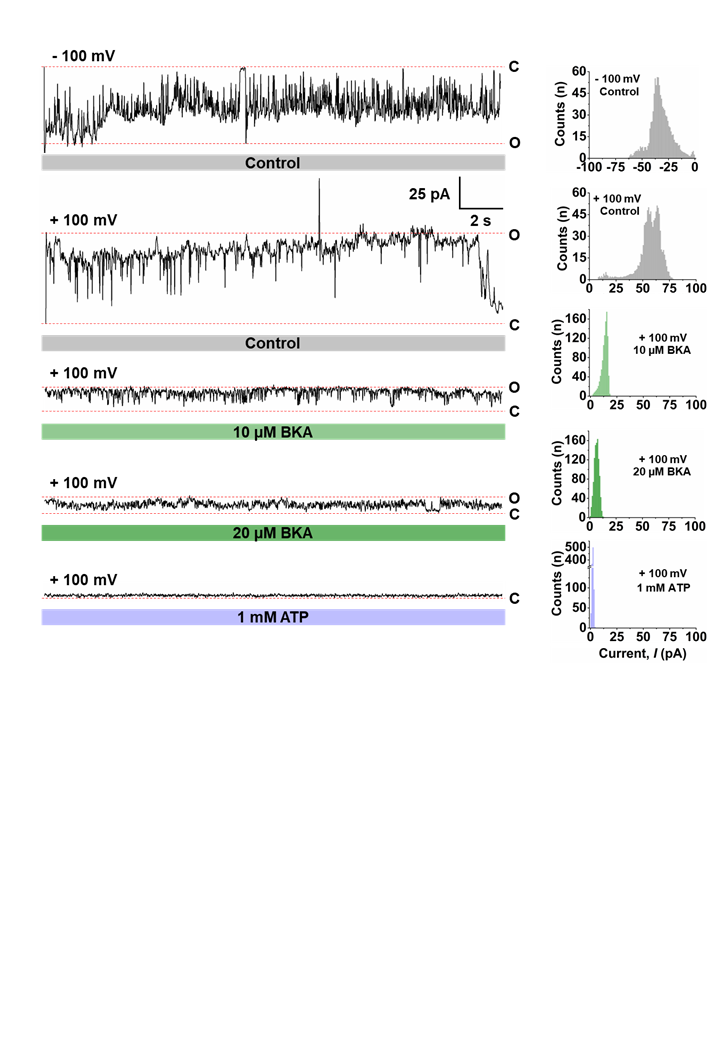

Supplement: Supplementary Figure 2 — Planar lipid bilayer electrophysiology recording of purified ANT. Truncated traces from continuous single-channel planar lipid bilayer recording of purified murine ANT at a holding potential of -100 mV and +100 mV. Traces show a decrease in channel current with increasing BKA concentration at +100 mV. Channel activity is completely inhibited with ATP (1 mM). Histograms shown on the right panel for each corresponding trace demonstrate a gradual decrease in channel current upon addition of BKA and ATP. ‘C’ and ‘O’ refer to the channel’s closed and open state, respectively. Signals were filtered at 5 kHz using the amplifier circuitry. [file Image2.tif]
